# Associations of breeding-bird abundance with climate vary among species and trait-based groups in southern California

**DOI:** 10.1371/journal.pone.0230614

**Published:** 2020-03-31

**Authors:** Frank A. Fogarty, Daniel R. Cayan, Laurel L. DeHaan, Erica Fleishman

**Affiliations:** 1 Department of Environmental Science and Policy, University of California, Davis, California, United States of America; 2 Climate, Atmospheric Sciences, and Physical Oceanography Division, Scripps Institution of Oceanography, University of California, San Diego, La Jolla, California, United States of America; San Diego Zoo Institute for Conservation Research, UNITED STATES

## Abstract

The responses of individuals and populations to climate change vary as functions of physiology, ecology, and plasticity. We investigated whether annual variation in seasonal temperature and precipitation was associated with relative abundances of breeding bird species at local and regional levels in southern California, USA, from 1968–2013. We tested our hypotheses that abundances were correlated positively with precipitation and negatively with temperature in this semiarid to arid region. We also examined whether responses to climate varied among groups of species with similar land-cover associations, nesting locations, and migratory patterns. We investigated relations between seasonal climate variables and the relative abundances of 41 species as estimated by the North American Breeding Bird Survey. Associations with climate variables varied among species. Results of models of species associated with arid scrublands or that nest on the ground strongly supported our hypotheses, whereas those of species associated with coniferous forests or that nest in cavities did not. Associations between climate variables and the abundances of other trait-based groups were diverse. Our results suggest that species in arid areas may be negatively affected by increased temperature and aridity, but species in nearby areas that are cooler and less arid may respond positively to those fluctuations in climate. Relations with climate variables can differ among similar species, and such knowledge may inform projections of future abundance trajectories and geographic ranges.

## Introduction

The effects of climate change on birds may manifest in many ways, including changes in geographic and elevational distributions [1, 2]; phenology, including the timing of migration and breeding initiation [3, 4]; community composition and species interactions [5, 6]; movement, including abbreviated or aborted migration [7]; physiology and behaviors related to foraging and breeding [8, 9, 10]; and abundance, including extirpation and colonization (for more-detailed reviews, see [11, 12]). These climate-related changes may interact with each other and with other environmental changes (e.g., land-cover change or fragmentation) in unexpected ways, especially when different but simultaneous environmental changes induce different responses in survival, reproduction, or distributions [13, 14]. Much research has examined whether climate change is associated with long-term changes in the phenology of birds (e.g., [15, 16]), and with realized or potential range shifts [17, 18]. Less research has examined whether long-term variation in abundances of birds are associated with long-term variation in weather or climate (but see [19]). Changes in abundance may reflect the cumulative effects of changes in survival, fecundity, phenology, and movement, and it often is difficult to discriminate among the effects of these processes.

Relations of bird populations to fluctuations in seasonal climate could inform projections of their responses to longer term-climate change. Such fluctuations, especially in precipitation, can influence survival and population dynamics in birds [20, 21]. Winter precipitation and, to a lesser extent, spring precipitation in the breeding range are expected to indirectly affect vital rates and abundances of breeding birds in temperate ecosystems. For example, in at least some cases, both adult survival and recruitment were positively correlated with high annual precipitation in the breeding range [22, 23]. In southern California, reproductive success of a passerine (Rufous-crowned Sparrow [*Aimophila ruficeps*]) was correlated positively with annual precipitation [24], and decreases in species richness and occupancy of breeding birds in the Mojave Desert over the last century were correlated with increased aridity, especially decreased precipitation [25]. In regions that are arid to semiarid, precipitation likely is correlated positively with primary productivity [26, 27, 28]. Primary productivity, in turn, likely is correlated positively with arthropod abundance and vegetation cover, which are resources for nesting birds [29]. Primary productivity in both the winter and breeding seasons correlates positively with population sizes of birds [30, 31], although extreme precipitation can negatively affect both reproductive success and adult survival [32, 33].

Air temperature also may affect the abundances of breeding birds. Previous work suggested that the effects of increases in temperature were inconsistent for a migratory passerine, documenting both increases in density and simultaneous reductions in adult survival in temperate regions [34]. Survival may decrease as aridity increases even among species adapted to warm and dry climates [35], and breeding activity of birds in arid regions may be constrained by temperature even if precipitation increases [36]. Increases in evapotranspiration may reduce primary productivity, although predicting these effects in semiarid systems may be difficult [37]. Increasing temperatures may lead to asynchronies between the emergence of invertebrate prey and avian breeding activity [38, 39].

We used long-term data from the North American Breeding Bird Survey in an arid to semi-arid region, southern California, to test three hypotheses that reflect the above knowledge and inference about mechanisms by which variation in season climate might affect breeding birds in such an environment. First, winter, and spring precipitation immediately preceding or overlapping with the breeding season in the breeding range are positively correlated with abundances of breeding birds. Second, winter and spring mean and maximum temperature immediately preceding or overlapping with the breeding season are negatively correlated with abundances of breeding birds. Third, responses to temperature and precipitation vary among groups of species that are based on land-cover associations, nest location, or migration patterns. Management plans of federal and state agencies in the United States often set targets related to land-cover types, reflecting their assumption that responses to management of land cover among species associated with that type will be similar [40, 41]. Nest location is relevant to climate responses because it can mediate exposure of adults and juveniles. Nest microclimate, which affects reproductive success, also is affected by nest location [42, 43]. Migration patterns affect the duration and timing of exposure to climate [44]. The responses of birds to climate variables and other environmental factors often are non-linear [45, 46, 47], so we also examined whether all of our hypothesized responses were linear or quadratic, and whether interactions between within-season linear variables (e.g., winter temperature * winter precipitation) were statistically significant.

## Materials and methods

### Bird data

**Fig 1:**
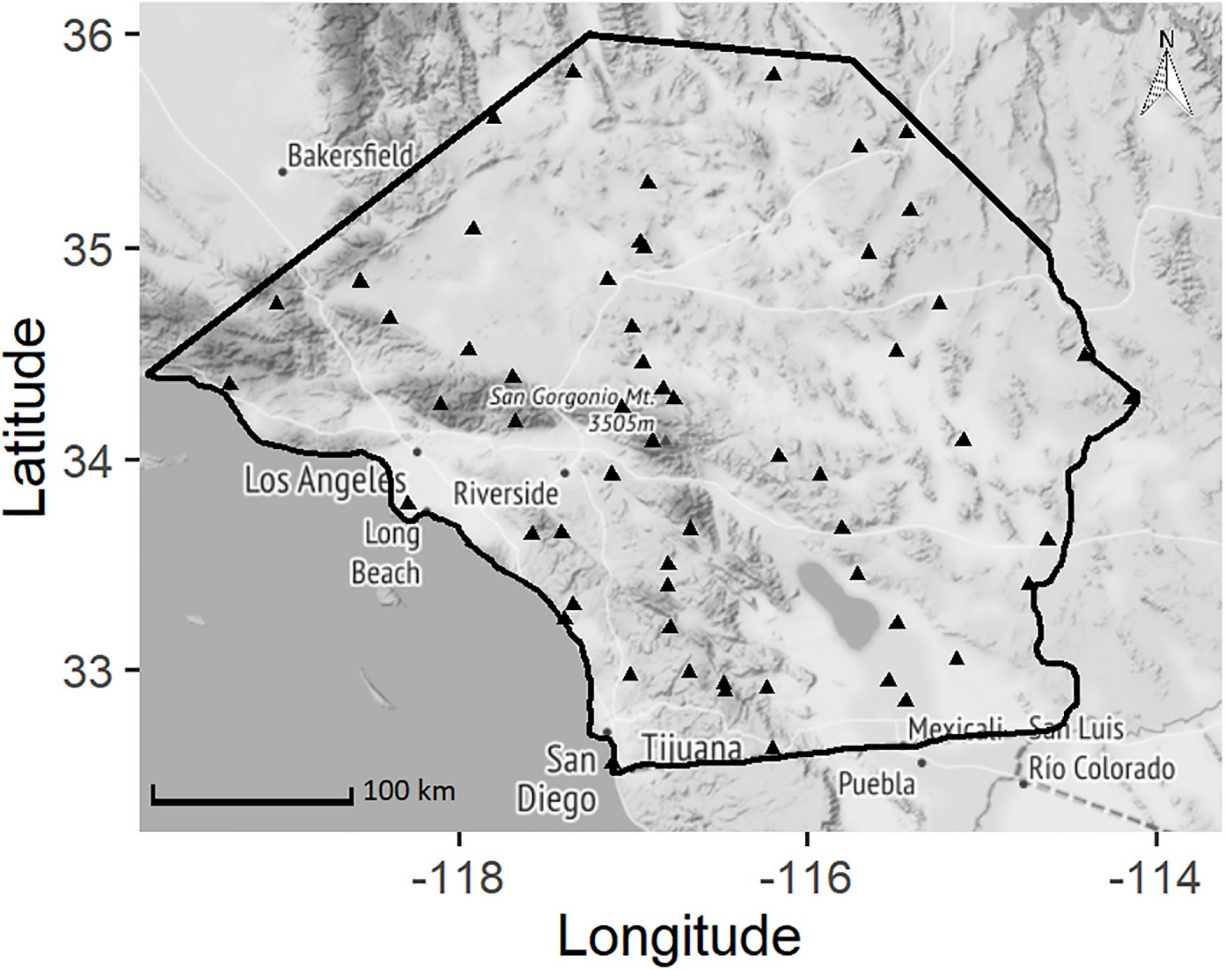
Starting points of Breeding Bird Survey routes that were included in our analysis (black triangles). Study area outlined in black. Map tiles by Stamen Design, under CC BY 3.0. Data by OpenStreetMap, under Open Data Commons Open Database License (ODbL) by the OpenStreetMap Foundation.

We used data from the North American Breeding Bird Survey (BBS) that were collected from 1968 through 2013 in four Level III ecoregions (an extent designed to coincide with regional environmental monitoring, assessment, reporting, and decision-making; [48, 49]): Southern / Central California Chaparral / Oak Woodlands, Southern California Mountains, Mojave Basin and Range, and Sonoran Basin and Range. We bounded our study area by the Transverse Ranges to the north, the Arizona border to the east, and the United States–Mexico border to the south (Fig 1). For simplicity, we often reference the area within our boundaries as southern California.

Our study area contained 60 BBS survey routes, the majority of which first were surveyed from 1968–1970. The BBS data primarily are collected to monitor long-term trends in the distribution and relative abundances of birds [50, 51, 52]. Routes are surveyed once per year during a window that coincides with the regional breeding season (April through June for most of the southwestern United States). These data reflect detections, and it is not possible to confirm that all records represent breeding individuals. Each route includes 50 point-count locations that are separated by approximately 800 m. Observers record all individual birds detected by sight or sound during a 3-minute survey (count) at each location. Not all routes are surveyed every year; routes in our study area were surveyed for a mean of 17.9 years (range 3–40). Although these data are collected by knowledgeable individuals, experience levels and familiarity with a given route may vary among observers and years. Because its single-visit data collection protocol does not allow for estimation of detection probability, the BBS data are biased to an unknown degree by imperfect detection [53]. Therefore, the BBS data are not absolute measures of abundance but indices of relative abundance. Furthermore, BBS routes are along roads and their locations are not fully randomized, which may bias estimates of abundance [54]. Therefore, our data and results should be interpreted as trends in relative abundance among BBS routes rather than absolute regional abundances. Nevertheless, there is some evidence that trends and responses derived from BBS data can be useful approximations of population-level trends of many species [52, 55], and few sources of data have the spatial and temporal extent of the BBS.

For each route in each year, we summed the reported abundance of each species at all 50 point-count locations. We did not model locations as nested within routes because coordinates of locations other than the starting points of the BBS routes, and BBS data at the resolution of locations for most of the time period neither are publicly available nor were possible for us to obtain. From the species detected by the BBS, we selected 41 taxa for which the BBS data included ≥ 1000 detections, which our experience suggested was the minimum necessary for likely model convergence and associated strong inference. These species collectively represented diverse life histories, land-cover associations, nest location, and migration patterns (S1 Table). All characterizations of these taxa herein refer to populations within our study area. In other parts of their ranges, the species may have different land-cover associations or migration patterns.

We defined land-cover classes with which the individual species of breeding birds typically are associated as arid scrubland, chaparral, coniferous forest, grassland, oak woodland, riparian, rocky slopes, or wetland [56, 57]. Descriptions of each land-cover class are in S2 Table. We classified 22 species as reliably associated with one of these land-cover classes and the remaining 19 species as associated with two or more classes. We classified species on the basis of four nest locations: cavities, shrubs (vegetation < 2 meters high), trees, and ground. Most of the 13 species that inhabit riparian land-cover also inhabit oak woodland; no species was associated only with riparian land-cover. Few species in the analysis regularly occupy wetlands, grasslands, or rocky slopes (two, three, and one species, respectively). Therefore, we did not examine associations between climate and the relative abundances of species associated with these four land-cover classes. Nor did we examine associations between climate and the relative abundances of species that nest in burrows, crevices, and reeds (one species each). Eight species are long-distance migrants. The remaining 33 species are either non-migratory or partially migratory (some individuals or populations within the study area are migratory). We classified partially migratory species as non-migratory because at least part of the population remains in our study area during winter. Non-migratory species may be affected directly by winter climate in the breeding range, whereas fully migratory species only can be affected indirectly (e.g., through the response of spring primary productivity to winter precipitation). We developed single-species models, then examined whether responses to climate were similar among species within groups defined on the basis of land-cover association, nest location, or migration pattern.

### Climate data

To investigate correlations between abundance and temperature and precipitation along the BBS routes, we used the [58] 6 km gridded monthly mean data that contained the start coordinates for each route. There may be some intra-route variability in weather, but because only the coordinates of the start points of the BBS routes are publicly available, it is difficult to account for such variability in our analyses. Because we are assessing relations between relative abundance indices and relative changes in weather variables at the route level, we do not think that intra-route variability likely had substantial effects on our results and inferences. Relative changes are likely to be highly correlated within the small area covered by the survey route, especially when aggregated over three months. From the [58] data we derived values of two climate attributes (precipitation and temperature) that we hypothesized to be associated with abundance for three seasons: winter (December-February) prior to the surveys, the season during which most precipitation occurs in the region and therefore that with the greatest effect on primary productivity; spring (March-May), the season during which most species are rearing young, and during which primary productivity remains high; and previous summer (June-August), a time period that did not directly relate to our hypotheses but relates to reproductive and pre-migratory activity the previous year.

We calculated Tair (mean daily surface air temperature) and Tmax (maximum daily surface air temperature) as an average of all of the days for each season, and we calculated mean daily precipitation for each season on the basis of daily precipitation (Table 1), for each BBS route. We tested whether each pair of variables was correlated before including them in our models and excluded variables for which correlations were > 0.7. Tair and Tmax were highly correlated (> 0.85) during both winter and spring, so we were unable to include both variables for the same season in our analyses. We included spring and previous summer Tmax (henceforth spring and previous summer temperature) in models of abundance because extremely warm days during spring or summer might increase heat-related mortality of birds [59, 60]. We included winter Tair in models of bird abundance because we hypothesized that it better captured the potential response of primary productivity and invertebrate abundance to temperature. Additionally, winter Tair (henceforth winter temperature) was slightly less correlated with all other climate variables than winter Tmax (largest correlations 0.17 versus -0.31, respectively). Given the high correlation between Tair and Tmax, it is unlikely that our choice of which of these two variables to include in models of abundance strongly affected the results. All other correlations of climate variables between seasons, and between precipitation and temperature, were ≤ 0.5.

**Table 1:** Mean values and range of climate variables included in our analysis. If a route was not surveyed in a given year, we did not include climate values for that year in our calculations. For the two route-level variables, we calculated variation as the range of values of the variable for each route across all years in which the route was surveyed, then averaged among all routes. The numbers in parentheses represent the individual routes with the least and greatest range. For the other two variables, the numbers in parentheses represent the routes with the lowest and highest annual means. Spring temperature was based on maximum daily values, whereas winter temperature was based on mean daily values. Routes with low annual variation generally were surveyed in few years, and therefore their true range of variation from 1968–2013 likely is underestimated.

	Winter	Spring
Route-level range in temperature among years, averaged among routes	3.53°C (least 1.14, greatest 5.44)	5.30°C (least 0.58, greatest 8.43)
Mean temperature among all routes and years	8.84°C (lowest -0.39, highest 14.04)	22.29°C (lowest 11.44, highest 30.51)
Route-level variation in precipitation among years, averaged among routes	4.25 mm/day (least 0.73, greatest 17.08)	2.20 mm/day (least 0.20, greatest 6.85)
Mean precipitation among all routes and years	1.80 mm/day (lowest 0.26, highest 4.98)	0.97 mm/day (lowest 0.07, highest 2.97)

### Associations of abundance with local precipitation and temperature

**Fig 2:**
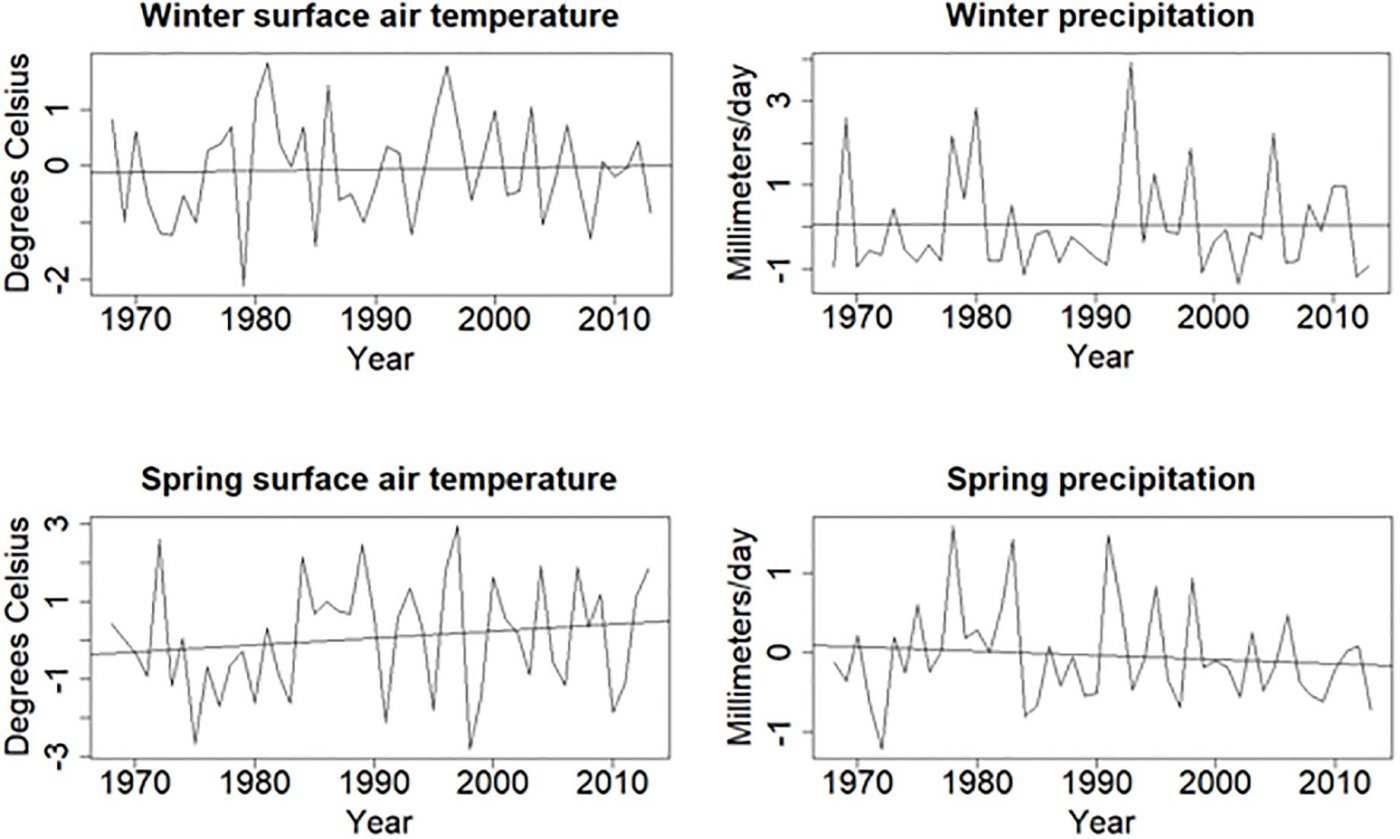
Average annual values of climate variables, relative to route means, for 1968 through 2013. We calculated the value for each year as the mean of all routes surveyed during that year relative to the mean among all routes and years included in the analysis. Trend lines were based on linear regression.

Values of a given climate variable differ among BBS routes in predictable ways regardless of annual climate variation. For example, seasonal and annual precipitation in any year differs appreciably between coastal and inland desert routes, and temperature differs between low- and high-elevation routes. Therefore, we modeled relative abundance as a function of standardized temperature and precipitation changes by route to account for regional climate gradients. We calculated the mean of each variable among years for each route. We then subtracted the mean from each annual value to calculate an offset. We included the offset values in the models. There also was considerable variation among years in climate variables. From 1968 through 2013, trends in spring temperature were slightly positive, trends in spring precipitation were slightly negative, and no trends in winter temperature and precipitation were apparent (Fig 2).

We fit generalized linear mixed models for individual species with the R package glmmTMB [61]. In the initial model for each species, we included both linear and quadratic fixed effects for the three seasons and two major attributes of climate (temperature and precipitation), and interaction effects of each climate attribute within a season (e.g., spring temperature * spring precipitation). We also included a fixed linear effect of calendar year to account for potential long-term trends in abundance that were not associated with annual climate, which previous analyses of BBS data suggested are common among species [53]. We included a random effect of survey route to account for differences in land cover and other environmental features among routes. Because the BBS count data likely were overdispersed with respect to the Poisson distribution, we examined the model fit of negative binomial distributions with variances that increase linearly as the mean increases (nbinom1) and with variances that increase quadratically as the mean increases (nbinom2). We selected the nbinom1 distribution on the basis of test runs for several species that yielded lower Akaike Information Criterion (AIC) scores than runs with the nbinom2 distribution. After fitting the initial model for each species with all covariates, we selected models by removing the fixed covariate with the highest *z*-score, refitting the new model and comparing its AIC score to that of the previous model, and continuing until the AIC score was minimized [62]. If the AIC scores of two models were within 2, we selected the model with the fewest covariates. We retained each linear covariate in the model if its quadratic form or an interaction term in which it was included still was present.

## Results

**Fig 3:**
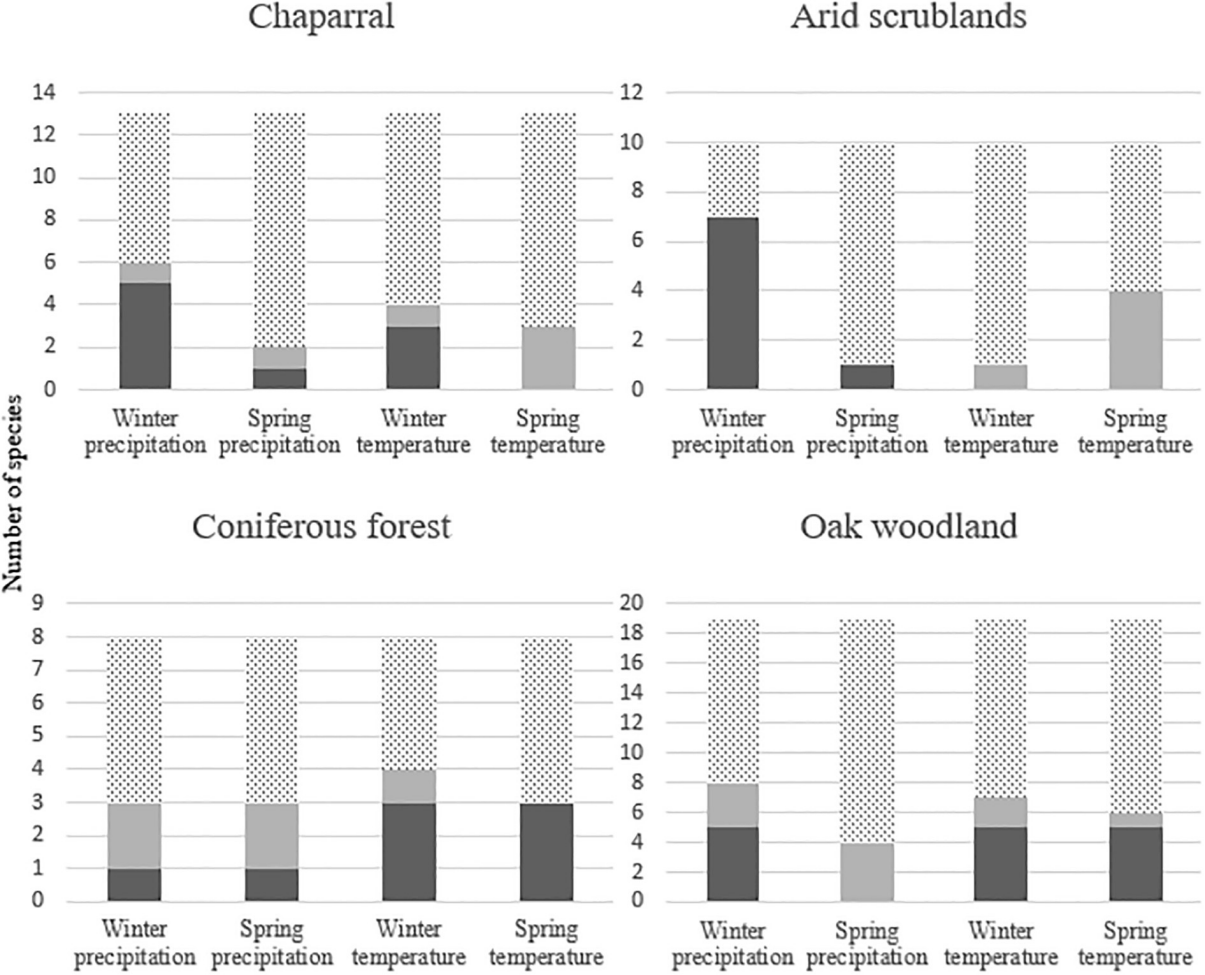
Significant associations between climate variables and abundances of species with different land-cover associations. Dark gray bars represent positive associations, pale gray bars represent negative associations, and dotted bars represent no association.

**Fig 4:**
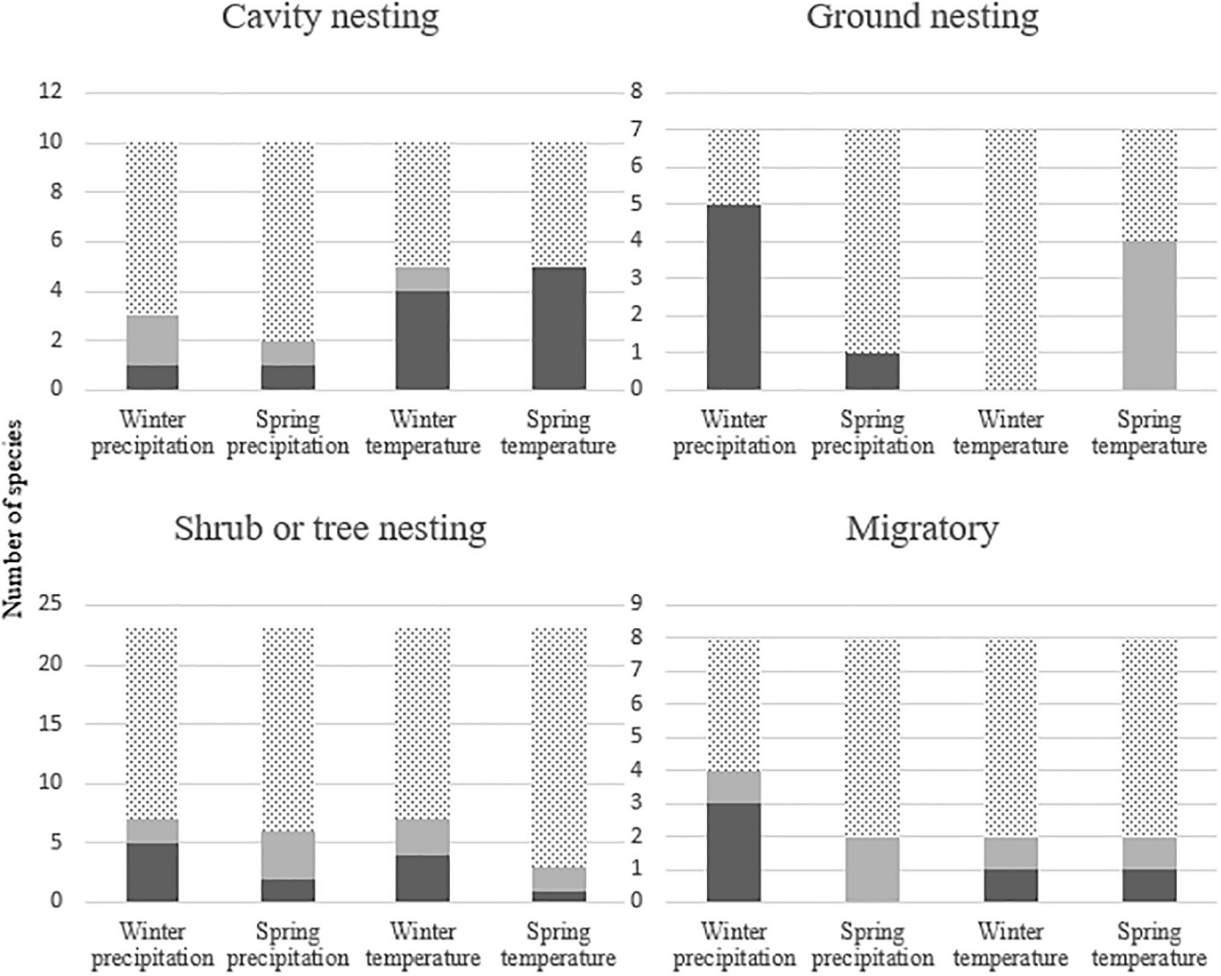
Significant associations between climate variables and abundances of species with different nest locations or that are long-distance migrants. Dark gray bars represent positive associations, pale gray bars represent negative associations, and dotted bars represent no association.

Although abundances of most species were significantly associated with at least one climate variable, the direction and pattern of these associations varied among species. In some cases, patterns emerged within groups of species that were based on land-cover association or nest location (Figs 3 & 4).

Relative abundances of 30 of 41 species were associated with either precipitation or temperature in winter or spring (S3 Table). Spring temperature, winter precipitation, winter temperature, and spring precipitation were significantly associated with the relative abundances of 14, 15, 12, and 10 species, respectively. The effect sizes of these significant associations varied considerably among species and climate variables. Positive associations with winter precipitation or negative associations with spring temperature accounted for nine of the ten largest effect sizes. For example, a one standard deviation increase in winter precipitation was associated with a 40.1% increase in the relative abundance of California Quail (*Callipepla californica*), and a one standard deviation increase in spring Tmax was associated with a 17.5% decrease in the relative abundance of Bell’s Sparrow (*Artemisiospiza belli*). The best model for each species and its coefficient estimates are in supporting information (S4 Table). Temperature and precipitation in the previous summer, which did not relate directly to our primary hypotheses, had fewer significant relations with relative abundance; details of these associations are in supporting information (S5 Table).

Associations of groups based on land cover, nest location, and migratory behavior with local climate varied. In the following, all references are to statistically significant associations. Relative abundances of eight and five of the 10 species associated with arid scrublands, respectively, were positively associated with precipitation and negatively associated with temperature (Fig 3). Relative abundances of five of the 13 species associated with chaparral were positively associated with winter precipitation. Of the eight species that often occupy coniferous forest, abundances of six were positively associated with temperature. Relative abundances of eight and six of the 19 species that inhabit oak woodland were associated, respectively, with winter precipitation and spring temperature. Relative abundances of five of 10 species that nest in tree cavities were positively associated with spring temperature, and five were associated with winter temperature (Fig 4). Relative abundances of five of seven species that nest on the ground were positively associated with winter precipitation, and four were negatively associated with spring temperature. Eleven of the 17 species that nest in shrubs also nest in trees, so we analyzed the 23 species in these two nest locations together. Relative abundances of no more than eight species were associated with a single climate variable. Relative abundances of six of the eight migratory species were associated with at least one climate variable, but there were no consistent trends, and no climate variable was associated with the abundances of more than four of these species.

## Discussion

We hypothesized that the relative abundance of breeding birds along BBS routes in southern California was positively correlated with winter and spring precipitation and negatively correlated with winter and spring temperature. Although winter precipitation, winter temperature, and spring temperature appeared to be the variables most consistently associated with relative abundances, the patterns of association were not clear or general. Indeed, only species associated with arid scrublands and species that nest on the ground consistently supported our hypotheses that abundances increased as precipitation increased or as temperature decreased in winter or spring. Associations with climate of groups of birds associated with oak woodland and chaparral generally supported our hypotheses, but there were multiple exceptions. Relations between climate variables and groups of birds that nest in shrubs and trees were inconsistent, whereas abundances of species that occur in coniferous forest or nest in cavities were positively correlated with temperature and negatively correlated with precipitation. Relative abundances of a limited number of species (11) were associated with spring precipitation, responses were both negative (six species) and positive (five species). Some spring precipitation may fall after breeding birds have established territories and begun breeding. Breeding Bird Surveys are conducted before most species fledge, so the counts do not reflect effects of spring precipitation on reproductive output. Abundances of six of the seven species that were most strongly related to local climate were associated with either winter precipitation or spring temperature.

Differences in the ecology of groups of birds that are associated with different land-cover types, nest locations, or migration patterns appear to contribute to the groups’ distinct associations with climate. For example, both arid scrublands and chaparral in our study region are dominated by shrubs and grow in areas with hot, dry summers. Arid scrublands generally have lower annual precipitation, cooler winters, and drier springs and summers than chaparral. Primary productivity (and likely arthropod abundance) in arid scrublands and chaparral is highly responsive to water availability [63, 64], which may explain why winter precipitation and spring temperature were positively and negatively correlated, respectively, with relative abundances of many species of birds. For example, previous work indicated that reproductive success of Loggerhead Shrikes (*Lanius ludovicianus*) varies positively with both winter and spring precipitation [65]. Alternatively, reductions in our measure of relative abundance could be related to detection. For instance, the call rate of Gambel’s Quail (*Callipepla gambelli*) increases in spring in response to higher winter precipitation [66]. The weaker relations between these climate variables and the relative abundances of chaparral-associated species may reflect that chaparral usually grows in areas with a milder and less arid climate than that of arid scrublands [67]. These patterns generally are consistent with hypotheses that increases in aridity will negatively affect birds associated with desert or semidesert shrublands.

In contrast to arid scrublands and chaparral species, the relative abundances of species associated with coniferous forest and oak woodland were not strongly associated with winter precipitation. Moreover, relative abundances of some of these species were positively associated with local winter and spring temperatures. Coniferous forests and oak woodlands tend to occur at higher elevations than shrublands, usually in areas that are cooler, less xeric, and have extensive shade [67]. Our results suggest that the abundances of bird species in coniferous forests and oak woodlands within our study area may not be limited by temperature maxima, at least at the levels recorded to date, although the upper critical temperatures of some species (e.g., Mountain Chickadee [*Poecile gambeli*]) are lower than those of related species that occur at lower elevations [68]. As a possible explanation, especially in cool, high-elevation coniferous forests, temperature and arthropod abundance may be positively correlated [69].

The abundances of cavity-nesting species generally were positively associated with local temperature. Previous work indicated that higher temperatures increase nestling survival in a cavity nesting species (Tree Swallows [*Tachycineta bicolor*]; [70]), that Mountain Chickadees actively select nest sites with warmer ambient temperatures [71], and that Northern Flickers (*Colaptes auratus*) in shaded areas shift their foraging to warmer sites with high ant abundances [72]. However, our results may be related to the fact that these species primarily occur in woodlands and coniferous forests in which trees are large enough for cavity excavation, and thus the response may be more closely related to our inferences about these land-cover types than to the species’ nesting requirements. Similarly, the association of abundances of ground-nesting species with cool winters and wet springs may be related to this group’s association with shrublands. In our study area, few bird species construct nests on the ground in coniferous forests or woodlands, perhaps because ground cover necessary for nest concealment is sparse in areas shaded by trees. Wet winters and cool springs in shrublands also may lead to increases in the cover of annual grasses and forbs, which help conceal nests and ground foraging, and may aid in temperature regulation of the nest [73]. Relative abundances of species that nest in shrubs and trees had weak associations with climate variables, likely because life history varies substantially among members of these groups. For example, nest locations of these species include low shrubs in open environments, small riparian trees in woodlands, and the canopy of large conifers. Species we classified as migratory are absent from the southwestern corner of the United States during winter, and thus any effects of winter climate in the region would be indirect and not act until the birds’ spring arrival. Life histories differ among migratory species, individuals of some species may not arrive on their breeding grounds until early April, and individuals use many sites across a large geographic area, much of it external to their breeding grounds, every year. The climate of most of those sites is not represented in our analysis.

Our work assumed no extensive climate- or human-driven land-cover changes along the BBS routes over the study period. Potential shifts in plant distributions [74, 17] and the interaction of land-use change and climate [75] may affect past and future associations between climate and abundances of birds. Global climate model projections suggest that changes in climate in our study area by 2100 will likely include a substantial temperature increase, a possible decrease in precipitation [76] and a more volatile form of daily and interannual precipitation variation [77, 78]. Our response variable, relative abundance, may be driven by conditions on wintering grounds, disease, human-subsidized predators, and land-use change. We recognize that changes in the distributions of these species, whether in response to changes in climate or to any other source of environmental or demographic variation, also could lead to apparent changes in abundance. Furthermore, climate may affect birds via complex interactions or time lags not captured by the variables we modeled. The absence of significant trends between many variables and relative abundance should not be interpreted as a lack of relation; shortcomings in the BBS design and data may obscure some relations. Nevertheless, the emergence of trends in relations between climate variables and abundance despite this potential complexity and confounding drivers suggests that annual climate may have a substantial effect on bird populations in southern California. Given the variation in associations between climate variables and relative abundance within a single region, even among closely related species, extrapolating expected responses across species and regions likely is not warranted and may lead to erroneous inferences. Projections of how trends in species abundance or distributions will change in response to climate change likely will be most accurate when they are based on empirical evidence about the responses of individual species to variation in climate.

## Supporting information

S1 Table Traits of the 41 species of breeding birds included in this analysis. (DOCX)

S2 Table Land-cover classes with which breeding birds in southern California are associated. (DOCX)

S3 Table Statistically significant associations between mean annual abundance and local climate variables for winter and spring derived from generalized linear models. -, negative association; +, positive association.
(DOCX)

S4 Table Model with the lowest AIC score for each species. If the AIC scores of two models were within 2, we selected the model with the fewest covariates. We retained each linear covariate in the model if its quadratic form or an interaction term in which it was included still was present. We further assessed model fit by plotting model predictions and their confidence intervals against the empirical data. We plotted statistically significant (p<0.05; indicated by an asterisk) associations with exponential or interaction effects for the range of -2 to 2 standard deviations to provide inference on the direction and magnitude of the association. Some significant, higher-order associations had equivocal associations with the response variable in these plots and were excluded. ‘Prev_Sum’ corresponds to climate variables from June to August of the previous year. Precip, precipitation; Tair, mean daily surface air temperature; Tmax, maximum daily surface air temperature. Pr, probability.
(DOCX)

S5 Table Statistically significant associations, derived from generalized linear models, between mean annual abundance and local climate variables for the previous summer (June-August). -, negative association; +, positive association.
(DOCX)
