# Supplementary material for: Associations of breeding-bird abundance with climate vary among species and trait-based groups in southern California
Source: PLoS One. 2020 Mar 31;15(3):e0230614. doi: 10.1371/journal.pone.0230614 (PMC7108724; doi:10.1371/journal.pone.0230614)
Supplement: S1 Table — (DOCX) [file pone.0230614.s001.docx]

**Supporting information**

**Table S1.** Traits of the 41 species of breeding birds included in this analysis.

| Species | Land-cover association | Nest location | Long-distance migrant |
| --- | --- | --- | --- |
| Mountain Quail (*Oreortyx pictus*) | chaparral | ground | no |
| California Quail (*Callipepla californica*) | arid scrublands, chaparral, oak woodland | ground | no |
| Gambel’s Quail (*Callipepla gambelii*) | arid scrublands | ground | no |
| Burrowing Owl (*Athene cunicularia*) | grassland | burrow | no |
| Anna’s Hummingbird (*Calypte anna*) | oak woodland, chaparral, riparian | shrub, tree | no |
| Acorn Woodpecker (*Melanerpes formicivorus*) | oak woodland | cavity | no |
| Nuttall’s Woodpecker (*Dryobates nuttallii*) | oak woodland | cavity | no |
| Northern Flicker (*Colaptes auratus*) | coniferous forest, oak woodland | cavity | no |
| Western Wood-Pewee (*Contopus sordidulus*) | oak woodland, riparian | tree | yes |
| Western Kingbird (*Tyrannus verticalis*) | grassland | shrub, tree | yes |
| Loggerhead Shrike (*Lanius ludovicianus*) | arid scrublands | shrub, tree | no |
| Steller’s Jay (*Cyanocitta stelleri*) | coniferous forest | tree | no |
| California Scrub-Jay (*Aphelocoma californica*) | oak woodland, chaparral, riparian | tree | no |
| Horned Lark (*Eremophila alpestris*) | arid scrublands | ground | no |
| Violet-green Swallow (*Tachycineta thalassina*) | coniferous forest | cavity | yes |
| Mountain Chickadee (*Poecile gambeli*) | coniferous forest | cavity | no |
| Oak Titmouse (*Baeolophus inornatus*) | oak woodland | cavity | no |
| Bushtit (*Psaltriparus minimus*) | oak woodland, chaparral, riparian | shrub, tree | no |
| White-breasted Nuthatch (*Sitta carolinensis*) | oak woodland | cavity | no |
| Pygmy Nuthatch (*Sitta pygmaea*) | coniferous forest | cavity | no |
| Rock Wren (*Salpinctes obsoletus*) | rocky slopes | crevice | no |
| House Wren (*Troglodytes aedon*) | oak woodland, riparian | cavity | no |
| Bewick’s Wren (*Thryomanes bewickii*) | chaparral, riparian | shrub, tree | no |
| Cactus Wren (*Campylorhynchus brunneicapillus*) | arid scrublands | shrub | no |
| Wrentit (*Chamaea fasciata*) | chaparral | shrub | no |
| Western Bluebird (*Sialia mexicana*) | coniferous forest, oak woodland | cavity | no |
| California Thrasher (*Toxostoma redivivum*) | chaparral | shrub, tree | no |
| LeConte’s Thrasher (*Toxostoma lecontei*) | arid scrublands | shrub | no |
| Black-chinned Sparrow (*Spizella atrogularis*) | arid scrublands, chaparral | ground, shrub | yes |
| Black-throated Sparrow (*Amphispiza bilineata*) | arid scrublands | ground | no |
| Bell’s Sparrow (*Artemisiospiza belli*) | arid scrublands, chaparral | shrub | yes |
| Song Sparrow (*Melospiza melodius*) | riparian, wetland | shrub, tree | no |
| California Towhee (*Melozone crissalis*) | oak woodland, chaparral, riparian | shrub, tree | no |
| Spotted Towhee (*Pipilo maculatus*) | oak woodland, chaparral | ground, shrub | no |
| Western Tanager (*Piranga ludoviciana*) | coniferous forest, oak woodland, riparian | tree | yes |
| Black-headed Grosbeak (*Pheucticus melanocephalus*) | coniferous forest, oak woodland, riparian | tree | yes |
| Lazuli Bunting (*Passerina amoena*) | arid scrublands, chaparral, oak woodland, riparian | shrub | yes |
| Western Meadowlark (*Sturnella neglecta*) | grassland | ground | no |
| Red-winged Blackbird (*Agelaius phoeniceus*) | wetland | reeds | no |
| House Finch (*Haemorhous mexicanus*) | oak woodland, riparian | shrub, tree, human-made | no |
| Lesser Goldfinch (*Spinus psaltria*) | oak woodland, riparian | shrub, tree | no |
