# Supplementary material for: Associations of breeding-bird abundance with climate vary among species and trait-based groups in southern California
Source: PLoS One. 2020 Mar 31;15(3):e0230614. doi: 10.1371/journal.pone.0230614 (PMC7108724; doi:10.1371/journal.pone.0230614)
Supplement: S2 Table — (DOCX) [file pone.0230614.s002.docx]

**Table S2.** Land-cover classes with which breeding birds in southern California are associated.

| Class | Characteristic plant species |
| --- | --- |
| Arid scrubland | *Larrea* spp.*, Atriplex* spp., *Fouquieria splendens*, *Yucca* spp. |
| Chaparral, including coastal sage scrub | *Adenostema fasciculatum*, *Ceanothus* spp., *Arctostaphylos* spp., *Quercus dumosa*, *Q. berberidifolia, Artemisia californica*, *Eriogonum* spp., *Salvia* spp. |
| Coniferous forest | *Abies concolor*, *Pinus* spp. |
| Grassland | Native and non-native Poaceae: *Avena* spp., *Bromus* spp., *Poa* spp., *Stipa* spp. |
| Oak woodland | *Quercus lobata*, *Q. agrifolia*, *Q. engelmannii*, *Heteromeles arbutifolia* |
| Riparian | *Populus* spp., *Platanus racemosa*, *Alnus rhombifolia*, *Salix* spp., *Juglans californica*, *Acer macrophyllum*, *Umbellularia californica* |
| Rocky slopes | Sparse vegetation |
| Wetland | In salt marsh or brackish areas: *Salicornia* spp., *Spartina* spp. In small freshwater marshes: *Polygonum* spp., *Scirpus* spp., *Typha latifolia*, *Juncus* spp., *Carex* spp. |
