# Supplementary material for: Associations of breeding-bird abundance with climate vary among species and trait-based groups in southern California
Source: PLoS One. 2020 Mar 31;15(3):e0230614. doi: 10.1371/journal.pone.0230614 (PMC7108724; doi:10.1371/journal.pone.0230614)
Supplement: S3 Table — -, negative association; +, positive association. (DOCX) [file pone.0230614.s003.docx]

**Table S3.** Statistically significant associations between mean annual abundance and local climate variables for winter and spring derived from generalized linear models. -, negative association; +, positive association.

| Species | Winter temperature | Winter precipitation | Spring temperature | Spring precipitation |
| --- | --- | --- | --- | --- |
| Mountain Quail |  | + | - |  |
| California Quail |  | + | - |  |
| Gambel’s Quail |  | + | - |  |
| Burrowing Owl |  |  |  |  |
| Anna’s Hummingbird |  |  |  |  |
| Acorn Woodpecker | + |  | + |  |
| Nuttall’s Woodpecker |  |  |  |  |
| Northern Flicker | + | - | + |  |
| Western Wood-Pewee |  |  | + |  |
| Western Kingbird |  |  |  |  |
| Loggerhead Shrike |  | + |  |  |
| Steller’s Jay |  |  |  |  |
| California Scrub-Jay | + |  |  | - |
| Horned Lark |  | + |  | + |
| Violet-green Swallow | + |  |  | - |
| Mountain Chickadee |  |  | + | + |
| Oak Titmouse |  |  |  |  |
| Bushtit | + | + |  |  |
| White-breasted Nuthatch |  | - | + |  |
| Pygmy Nuthatch | + |  |  |  |
| Rock Wren |  |  | + | - |
| House Wren | - | + |  |  |
| Bewick’s Wren | + |  |  | + |
| Cactus Wren |  |  |  |  |
| Wrentit |  | - |  |  |
| Western Bluebird |  |  | + |  |
| California Thrasher | - |  |  |  |
| LeConte’s Thrasher | - |  |  |  |
| Black-chinned Sparrow |  |  |  |  |
| Black-throated Sparrow |  | + | - |  |
| Bell’s Sparrow |  | + | - |  |
| Song Sparrow |  |  | - | + |
| California Towhee |  |  |  |  |
| Spotted Towhee |  |  |  |  |
| Western Tanager | - | + |  |  |
| Black-headed Grosbeak |  | - |  | - |
| Lazuli Bunting |  | + |  |  |
| Western Meadowlark |  |  |  |  |
| Red-winged Blackbird |  |  | - |  |
| House Finch |  |  |  | - |
| Lesser Goldfinch | + |  |  | - |
