# Supplementary material for: Associations of breeding-bird abundance with climate vary among species and trait-based groups in southern California
Source: PLoS One. 2020 Mar 31;15(3):e0230614. doi: 10.1371/journal.pone.0230614 (PMC7108724; doi:10.1371/journal.pone.0230614)
Supplement: S4 Table — If the AIC scores of two models were within 2, we selected the model with the fewest covariates. We retained each linear covariate in the model if its quadratic form or an interaction term in which it was included still was present. We further assessed model fit by plotting model predictions and their confidence intervals against the empirical data. We plotted statistically significant (p<0.05; indicated by an asterisk) associations with exponential or interaction effects for the range of -2 to 2 standard deviations to provide inference on the direction and magnitude of the association. Some significant, higher-order associations had equivocal associations with the response variable in these plots and were excluded. ‘Prev_Sum’ corresponds to climate variables from June to August of the previous year. Precip, precipitation; Tair, mean daily surface air temperature; Tmax, maximum daily surface air temperature. Pr, probability. (DOCX) [file pone.0230614.s004.docx]

**Table S4.** Model with the lowest AIC score for each species. If the AIC scores of two models were within 2, we selected the model with the fewest covariates. We retained each linear covariate in the model if its quadratic form or an interaction term in which it was included still was present. We further assessed model fit by plotting model predictions and their confidence intervals against the empirical data. We plotted statistically significant (p<0.05; indicated by an asterisk) associations with exponential or interaction effects for the range of -2 to 2 standard deviations to provide inference on the direction and magnitude of the association. Some significant, higher-order associations had equivocal associations with the response variable in these plots and were excluded. ‘Prev_Sum’ corresponds to climate variables from June to August of the previous year. Precip, precipitation; Tair, mean daily surface air temperature; Tmax, maximum daily surface air temperature. Pr, probability.

*Mountain Quail*

Estimate Standard Error z value Pr(>|z|)

Intercept -5.74 1.675 -3.427 0.001 *

Prev_Sum_Tmax^2 0.055 0.021 2.633 0.008 *

Winter_Precip 0.087 0.028 3.144 0.001 *

Spring_Tmax -0.343 0.051 -6.713 0.000 *

Prev_Sum_Tmax 0.119 0.039 3.086 0.002 *

Year -0.149 0.057 -2.624 0.009 *

*California Quail*

Estimate Standard Error z value Pr(>|z|)

Intercept -0.718 0.544 -1.320 0.187

Prev_Sum_Tmax^2 0.056 0.019 2.936 0.003 *

Winter_Precip^2 -0.020 0.014 -1.475 0.140

Spring_Precip 0.042 0.029 1.467 0.142

Spring_Tmax -0.161 0.040 -4.052 0.000 *

Prev_Sum_Tmax -0.037 0.029 -1.298 0.194

Year 0.083 0.034 2.430 0.015 *

Winter_Precip 0.160 0.040 4.001 0.000 *

*Gambel’s Quail*

Estimate Standard Error z value Pr(>|z|

Intercept -1.429 0.612 -2.335 0.020 *

Prev_Sum_Precip^2 -0.025 0.021 -1.164 0.244

Spring_Precip^2 -0.168 0.139 -1.215 0.224

Spring_Tmax^2 -0.067 0.048 -1.391 0.164

Winter_Precip^2 -0.084 0.073 -1.149 0.251

Winter_Precip 0.438 0.140 3.119 0.002 *

Spring_Precip 0.187 0.171 1.095 0.274

Spring_Tmax -0.143 0.055 -2.570 0.010 *

Prev_Sum_Precip 0.160 0.075 2.149 0.032 *

Prev_Sum_Tmax 0.098 0.059 1.659 0.097

Year 0.186 0.062 3.019 0.002 *

*Burrowing Owl*

Estimate Standard Error z value Pr(>|z|)

Intercept -3.892 1.052 -3.699 0.000 *

Winter_Tair 0.086 0.056 1.539 0.124

Prev_Sum_Precip -0.079 0.049 -1.600 0.110

Prev_Sum_Tmax -0.069 0.064 -1.077 0.282

Year 0.122 0.059 2.067 0.039 *

*Anna’s Hummingbird*

Estimate Standard Error z value Pr(>|z|)

Intercept -1.254 0.443 -2.829 0.005 *

Spring_Precip^2 -0.038 0.018 -2.161 0.031 *

Winter_Precip 0.044 0.027 1.647 0.010

Spring_Precip 0.029 0.045 0.640 0.522

Spring_Tmax -0.060 0.057 -1.058 0.290

Year 0.181 0.050 3.646 0.000 *

Spring_Precip:Spring_Tmax -0.066 0.038 -1.761 0.078

*Acorn Woodpecker*

Estimate Standard Error z value Pr(>|z|)

Intercept -5.572 1.989 -2.801 0.005 *

Prev_Sum_Tmax^2 0.043 0.017 2.467 0.014*

Winter_Tair^2 0.065 0.029 2.229 0.026 *

Winter_Precip -0.038 0.024 -1.619 0.106

Winter_Tair 0.084 0.034 2.483 0.013 *

Spring_Tmax 0.117 0.034 3.397 0.001 *

Prev_Sum_Tmax -0.041 0.030 -1.366 0.172

Winter_Precip:Winter_Tair 0.036 0.021 1.696 0.090

*Nuttall’s Woodpecker*

Estimate Standard Error z value Pr(>|z|)

Intercept -3.232 0.823 -3.927 0.000 *

Winter_Tair^2 0.115 0.036 3.225 0.001 *

Winter_Precip -0.064 0.034 -1.851 0.064

Winter_Tair -0.037 0.045 -0.823 0.411

Year 0.391 0.052 7.508 0.000 *

*Northern Flicker*

Estimate Standard Error z value Pr(>|z|)

Intercept -1.245 0.413 -3.012 0.003 *

Prev_Sum_Precip^2 -0.026 0.013 -2.023 0.043 *

Winter_Precip^2 0.032 0.010 3.255 0.001 *

Winter_Tair^2 0.061 0.026 2.360 0.018 *

Winter_Precip -0.153 0.038 -4.037 0.000 *

Winter_Tair 0.032 0.033 0.979 0.328

Spring_Tmax 0.090 0.034 2.610 0.009 *

Prev_Sum_Precip 0.088 0.045 1.946 0.052

Year -0.115 0.044 -2.606 0.009 *

Winter_Precip:Winter_Tair 0.054 0.022 2.455 0.014 *

*Western Wood-Pewee*

Estimate Standard Error z value Pr(>|z|)

Intercept -1.529 0.567 -2.695 0.007 *

Spring_Precip^2 -0.012 0.008 -1.528 0.126

Winter_Precip -0.031 0.019 -1.606 0.108

Spring_Precip 0.032 0.031 1.040 0.298

Spring_Tmax 0.078 0.040 1.980 0.048 *

Year -0.164 0.040 -4.072 0.000 *

*Western Kingbird*

Estimate Standard Error z value Pr(>|z|)

Intercept 0.658 0.193 3.413 0.001 *

Spring_Tmax 0.084 0.028 3.064 0.002 *

Prev_Sum_Precip 0.048 0.025 1.891 0.059

Prev_Sum_Tmax -0.048 0.029 -1.641 0.101

Year 0.058 0.031 1.889 0.059

*Loggerhead Shrike*

Estimate Standard Error z value Pr(>|z|)

Intercept 0.062 0.244 0.255 0.799

Spring_Precip^2 -0.061 0.040 -1.516 0.129

Spring_Tmax^2 -0.083 0.037 -2.250 0.024 *

Winter_Precip^2 -0.064 0.036 -1.759 0.079

Winter_Precip 0.210 0.072 2.917 0.004 *

Spring_Precip -0.009 0.069 -0.131 0.896

Spring_Tmax -0.041 0.040 -1.031 0.303

Year -0.336 0.043 -7.819 0.000 *

Spring_Precip:Spring_Tmax -0.152 0.077 -1.979 0.048 *

*Steller’s Jay*

Estimate Standard Error z value Pr(>|z|)

Intercept -8.657 1.310 -6.608 0.000 *

Spring_Tmax^2 -0.037 0.022 -1.700 0.089

Spring_Tmax 0.049 0.027 1.806 0.071

*California Scrub-Jay*

Estimate Standard Error z value Pr(>|z|)

Intercept -1.100 0.705 -1.560 0.119

Prev_Sum_Precip^2 -0.028 0.013 -2.179 0.029 *

Prev_Sum_Tmax^2 0.024 0.011 2.191 0.028 *

Winter_Tair 0.061 0.020 2.996 0.003 *

Spring_Precip -0.039 0.014 -2.755 0.006 *

Prev_Sum_Precip 0.048 0.033 1.432 0.152

Prev_Sum_Tmax -0.010 0.018 -0.565 0.572

*Horned Lark*

Estimate Standard Error z value Pr(>|z|)

Intercept 1.553 0.284 5.475 0.000 *

Prev_Sum_Tmax^2 -0.047 0.027 -1.723 0.085

Spring_Precip^2 -0.041 0.025 -1.672 0.094

Winter_Precip^2 -0.069 0.035 -1.973 0.048 *

Winter_Precip 0.222 0.064 3.474 0.001 *

Winter_Tair 0.048 0.030 1.601 0.109

Spring_Precip 0.135 0.056 2.394 0.017 *

Prev_Sum_Tmax 0.004 0.034 0.114 0.909

Year -0.157 0.043 -3.672 0.000 *

*Violet-green Swallow*

Estimate Standard Error z value Pr(>|z|)

Intercept -2.905 1.078 -2.695 0.007 *

Prev_Sum_Tmax^2 -0.018 0.013 -1.412 0.158

Spring_Tmax^2 -0.048 0.034 -1.430 0.153

Winter_Tair^2 0.053 0.022 2.420 0.016 *

Winter_Tair 0.079 0.032 2.495 0.013 *

Spring_Precip -0.075 0.037 -2.018 0.044 *

Spring_Tmax -0.012 0.050 -0.251 0.802

Prev_Sum_Tmax 0.052 0.026 2.022 0.043 *

Year -0.461 0.057 -8.127 0.000 *

Spring_Precip:Spring_Tmax -0.052 0.033 -1.600 0.110

*Mountain Chickadee*

Estimate Standard Error z value Pr(>|z|)

Intercept -7.369 1.325 -5.561 0.000 *

Spring_Precip^2 -0.034 0.013 -2.635 0.008 *

Winter_Tair 0.047 0.033 1.432 0.152

Spring_Precip 0.067 0.038 1.734 0.083

Spring_Tmax 0.110 0.041 2.665 0.008 *

Spring_Precip:Spring_Tmax -0.053 0.028 -1.886 0.059

*Oak Titmouse*

Estimate Standard Error z value Pr(>|z|)

Intercept -2.638 0.948 -2.783 0.005 *

Winter_Tair^2 0.069 0.031 2.221 0.026 *

Winter_Tair -0.029 0.037 -0.793 0.428

Year -0.067 0.040 -1.661 0.097

*Bushtit*

Estimate Standard Error z value Pr(>|z|)

Intercept -0.777 0.578 -1.346 0.178

Prev_Sum_Precip^2 -0.048 0.033 -1.483 0.138

Spring_Precip^2 -0.063 0.026 -2.458 0.014 *

Spring_Tmax^2 -0.100 0.065 -1.553 0.121

Winter_Tair^2 -0.077 0.043 -1.789 0.074

Winter_Precip 0.098 0.034 2.902 0.004 *

Winter_Tair 0.107 0.050 2.148 0.032 *

Spring_Precip -0.037 0.057 -0.646 0.518

Spring_Tmax -0.062 0.065 -0.959 0.338

Prev_Sum_Precip 0.024 0.080 0.304 0.761

Prev_Sum_Tmax -0.085 0.046 -1.870 0.062

Spring_Precip:Spring_Tmax -0.147 0.070 -2.097 0.036 *

*White-breasted Nuthatch*

Estimate Standard Error z value Pr(>|z|)

Intercept -4.251 1.276 -3.331 0.001 *

Winter_Precip -0.065 0.033 -1.980 0.048 *

Spring_Tmax 0.131 0.046 2.852 0.004 *

Year 0.192 0.058 3.309 0.001 *

*Pygmy Nuthatch*

Estimate Standard Error z value Pr(>|z|)

Intercept -8.308 1.411 -5.887 0.000 *

Winter_Tair 0.221 0.065 3.420 0.001 *

Spring_Precip 0.102 0.073 1.396 0.163

Spring_Tmax 0.163 0.092 1.770 0.077

Prev_Sum_Precip -0.018 0.042 -0.427 0.670

Prev_Sum_Tmax 0.032 0.049 0.646 0.518

Year -0.265 0.103 -2.580 0.010 *

Spring_Precip:Spring_Tmax 0.071 0.045 1.586 0.113

Prev_Sum_Precip:Prev_Sum_Tmax 0.068 0.030 2.258 0.024 *

*Rock Wren*

Estimate Standard Error z value Pr(>|z|)

Intercept -0.894 0.211 -4.241 0.000 *

Winter_Precip^2 0.034 0.019 1.816 0.069

Winter_Precip -0.085 0.075 -1.140 0.255

Spring_Precip -0.166 0.065 -2.532 0.011 *

Spring_Tmax -0.317 0.057 -5.601 0.000 *

Year 0.134 0.063 2.138 0.033 *

Spring_Precip:Spring_Tmax -0.104 0.046 -2.282 0.023 *

*House Wren*

Estimate Standard Error z value Pr(>|z|)

Intercept -2.183 0.922 -2.367 0.018 *

Spring_Tmax^2 -0.084 0.049 -1.714 0.086

Winter_Precip^2 -0.018 0.010 -1.775 0.076

Winter_Precip 0.134 0.042 3.185 0.001 *

Winter_Tair -0.032 0.044 -0.741 0.459

Spring_Precip -0.048 0.040 -1.189 0.234

Spring_Tmax -0.004 0.053 -0.070 0.944

Winter_Precip:Winter_Tair -0.057 0.024 -2.434 0.015 *

Spring_Precip:Spring_Tmax -0.064 0.039 -1.623 0.105

*Bewick’s Wren*

Estimate Standard Error z value Pr(>|z|)

Intercept 0.360 0.300 1.202 0.229

Prev_Sum_Precip^2 -0.034 0.022 -1.577 0.115

Winter_Tair^2 0.060 0.028 2.173 0.030 *

Winter_Tair 0.042 0.034 1.206 0.228

Spring_Precip 0.060 0.026 2.331 0.020 *

Prev_Sum_Precip 0.079 0.058 1.349 0.177

Prev_Sum_Tmax -0.074 0.033 -2.267 0.023 *

*Cactus Wren*

Estimate Standard Error z value Pr(>|z|)

Intercept -0.723 0.405 -1.786 0.074

Prev_Sum_Precip 0.127 0.028 4.552 0.000 *

Prev_Sum_Tmax -0.094 0.038 -2.457 0.014 *

Year 0.067 0.039 1.725 0.085

*Wrentit*

Estimate Standard Error z value Pr(>|z|)

Intercept -4.127 2.434 -1.696 0.090

Winter_Precip^2 0.024 0.008 3.063 0.002 *

Winter_Precip -0.106 0.031 -3.451 0.001 *

Year -0.085 0.034 -2.478 0.013 *

*Western Bluebird*

Estimate Standard Error z value Pr(>|z|)

Intercept -3.158 1.150 -2.745 0.006 *

Spring_Tmax 0.091 0.034 2.688 0.007 *

Prev_Sum_Tmax 0.057 0.029 1.988 0.047 *

*California Thrasher*

Estimate Standard Error z value Pr(>|z|)

Intercept -2.439 0.803 -3.038 0.002 *

Winter_Precip -0.030 0.027 -1.101 0.271

Winter_Tair -0.096 0.036 -2.677 0.007 *

Year -0.110 0.040 -2.772 0.006 *

Winter_Precip:Winter_Tair 0.046 0.024 1.953 0.051

*LeConte’s Thrasher*

Estimate Standard Error z value Pr(>|z|)

Intercept -3.745 1.080 -3.468 0.001 *

Prev_Sum_Precip^2 0.092 0.034 2.681 0.007 *

Prev_Sum_Tmax^2 0.101 0.058 1.733 0.083

Winter_Precip^2 -0.373 0.163 -2.295 0.022 *

Winter_Precip 0.239 0.147 1.628 0.103

Winter_Tair -0.107 0.051 -2.114 0.035 *

Prev_Sum_Precip -0.216 0.103 -2.103 0.035 *

Prev_Sum_Tmax -0.103 0.061 -1.686 0.092

Year -0.383 0.069 -5.586 0.000 *

Prev_Sum_Precip:Prev_Sum_Tmax 0.186 0.092 2.006 0.045 *

*Black-chinned Sparrow*

Estimate Standard Error z value Pr(>|z|)

Intercept -3.000 1.222 -2.452 0.014 *

Winter_Precip -0.025 0.031 -0.826 0.409

Winter_Tair -0.073 0.045 -1.612 0.107

Year -0.306 0.056 -5.430 0.000 *

Winter_Precip:Winter_Tair -0.042 0.026 -1.607 0.108

*Black-throated Sparrow*

Estimate Standard Error z value Pr(>|z|)

Intercept 0.030 0.453 0.066 0.947

Prev_Sum_Precip^2 -0.050 0.019 -2.647 0.008 *

Spring_Tmax^2 0.039 0.024 1.605 0.108

Winter_Precip^2 -0.130 0.053 -2.470 0.014 *

Winter_Tair^2 0.050 0.021 2.446 0.014 *

Winter_Precip 0.290 0.075 3.878 0.000 *

Winter_Tair 0.019 0.027 0.702 0.483

Spring_Tmax -0.068 0.028 -2.449 0.0143 *

Prev_Sum_Precip 0.095 0.050 1.914 0.056

Prev_Sum_Tmax 0.024 0.035 0.696 0.487

Winter_Precip:Winter_Tair -0.092 0.060 -1.539 0.124

Prev_Sum_Precip:Prev_Sum_Tmax -0.122 0.039 -3.131 0.002 *

*Bell’s Sparrow*

Estimate Standard Error z value Pr(>|z|)

Intercept -1.790 0.473 -3.786 0.00 *

Prev_Sum_Tmax^2 0.093 0.041 2.290 0.022 *

Winter_Precip^2 -0.034 0.024 -1.427 0.154

Winter_Tair^2 0.090 0.040 2.240 0.025 *

Winter_Precip 0.213 0.084 2.540 0.011 *

Winter_Tair 0.040 0.057 0.710 0.478

Spring_Tmax -0.218 0.061 -3.571 0.000 *

Prev_Sum_Precip 0.107 0.072 1.493 0.135

Prev_Sum_Tmax -0.086 0.060 -1.438 0.150

Year 0.244 0.075 3.244 0.001 *

Winter_Precip:Winter_Tair -0.075 0.052 -1.460 0.144

*Song Sparrow*

Estimate Standard Error z value Pr(>|z|)

Intercept -0.841 0.451 -1.866 0.0620

Spring_Tmax^2 -0.069 0.041 -1.695 0.090

Spring_Precip 0.129 0.038 3.391 0.001 *

Spring_Tmax 0.169 0.059 2.857 0.004 *

Prev_Sum_Precip 0.079 0.040 1.944 0.052

Prev_Sum_Tmax 0.156 0.033 4.752 0.000 *

Year 0.135 0.051 2.629 0.009 *

*California Towhee*

Estimate Standard Error z value Pr(>|z|)

Intercept -2.326 1.221 -1.905 0.057

Prev_Sum_Tmax^2 0.021 0.014 1.507 0.132

Spring_Tmax^2 -0.035 0.022 -1.565 0.118

Spring_Tmax -0.038 0.028 -1.353 0.176

Prev_Sum_Precip -0.016 0.028 -0.564 0.573

Prev_Sum_Tmax -0.019 0.023 -0.819 0.413

*Spotted Towhee*

Estimate Standard Error z value Pr(>|z|)

Intercept -0.971 0.802 -1.210 0.226

Spring_Tmax^2 -0.031 0.022 -1.398 0.162

Spring_Tmax 0.041 0.027 1.529 0.126

Prev_Sum_Tmax -0.052 0.025 -2.093 0.036 *

*Western Tanager*

Estimate Standard Error z value Pr(>|z|)

Intercept -2.483 0.586 -4.234 0.000 *

Winter_Tair -0.099 0.038 -2.586 0.010 *

Spring_Tmax 0.100 0.037 2.663 0.008 *

Prev_Sum_Tmax 0.098 0.031 3.207 0.001 *

Year -0.307 0.057 -5.393 0.000 *

*Black-headed Grosbeak*

Estimate Standard Error z value Pr(>|z|)

Intercept -0.562 0.401 -1.403 0.161

Spring_Precip^2 0.016 0.007 2.253 0.0242 *

Winter_Precip -0.061 0.019 -3.139 0.002 *

Winter_Tair -0.043 0.029 -1.486 0.137

Spring_Precip -0.048 0.021 -2.245 0.025 *

Year -0.266 0.035 -7.657 0.000 *

*Lazuli Bunting*

Estimate Standard Error z value Pr(>|z|)

Intercept -2.087 0.568 -3.677 0.000 *

Winter_Precip^2 -0.039 0.014 -2.886 0.004 *

Winter_Tair^2 0.136 0.048 2.846 0.004 *

Winter_Precip 0.174 0.056 3.126 0.002 *

Winter_Tair -0.001 0.057 -0.014 0.989

Spring_Precip -0.078 0.050 -1.568 0.117

Spring_Tmax -0.123 0.073 -1.699 0.089

*Western Meadowlark*

Estimate Standard Error z value Pr(>|z|)

Intercept 0.971 0.299 3.248 0.001 *

Spring_Precip 0.057 0.026 2.142 0.032 *

Prev_Sum_Precip 0.028 0.029 0.957 0.339

Prev_Sum_Tmax 0.012 0.030 0.415 0.678

Year -0.071 0.034 -2.096 0.036 *

Prev_Sum_Precip:Prev_Sum_Tmax -0.086 0.039 -2.206 0.027 *

*Red-winged Blackbird*

Estimate Standard Error z value Pr(>|z|)

Intercept 1.757 0.424 4.142 0.000 *

Prev_Sum_Tmax^2 0.047 0.027 1.789 0.074

Winter_Precip -0.002 0.046 -0.043 0.966

Winter_Tair -0.042 0.034 -1.251 0.211

Spring_Tmax -0.104 0.033 -3.126 0.002 *

Prev_Sum_Tmax 0.019 0.033 0.559 0.576

Year -0.076 0.038 -1.987 0.047 *

Winter_Precip:Winter_Tair 0.082 0.045 1.834 0.067

*House Finch*

Estimate Standard Error z value Pr(>|z|)

Intercept 2.813 0.169 16.639 0.000 *

Prev_Sum_Tmax^2 0.024 0.013 1.835 0.067

Spring_Tmax^2 0.037 0.022 1.662 0.096

Winter_Tair -0.033 0.020 -1.596 0.111

Spring_Precip -0.080 0.024 -3.357 0.001 *

Spring_Tmax -0.022 0.025 -0.889 0.374

Prev_Sum_Tmax 0.001 0.019 0.056 0.955

Year -0.065 0.027 -2.462 0.014 *

Spring_Precip:Spring_Tmax 0.027 0.028 0.972 0.331

*Lesser Goldfinch*

Estimate Standard Error z value Pr(>|z|)

Intercept 0.307 0.313 0.981 0.327

Winter_Tair^2 0.061 0.033 1.839 0.066

Winter_Tair 0.104 0.039 2.679 0.007 *

Spring_Precip -0.073 0.030 -2.402 0.016 *

Prev_Sum_Precip -0.078 0.045 -1.718 0.086

Prev_Sum_Tmax -0.099 0.037 -2.684 0.007 *
